# The Cysteine Protease CfAtg4 Interacts with CfAtg8 to Govern the Growth, Autophagy and Pathogenicity of *Colletotrichum fructicola*

**DOI:** 10.3390/jof10060431

**Published:** 2024-06-18

**Authors:** Shufeng Guo, Shengpei Zhang

**Affiliations:** 1 College of Forestry, Central South University of Forestry and Technology, Changsha 410004, China; gsf120312361@163.com; 2 Key Laboratory of Forest Bio-Resources and Integrated Pest Management for Higher Education in Hunan Province, Changsha 410004, China; 3 Hunan Provincial Key Laboratory for Control of Forest Diseases and Pests, Changsha 410004, China

**Keywords:** autophagy, CfAtg4, cleavage, pathogenicity, *C. fructicola*

## Abstract

*Camellia oleifera* is a native woody oil plant in southern China and is infected with anthracnose wherever it is grown. We previously identified *Colletotrichum fructicola* as the major causal agent of anthracnose on *C. oleifera* and found that CfAtg8 regulates the pathogenicity and development of *C. fructicola*. Here, we revealed that CfAtg4 interacts with CfAtg8, contributing to the formation of autophagosomes. The CfAtg8^1–160^ allele, which only contains 1–160 amino acids of the CfAtg8, partially recovered the autophagosome numbers and autophagy defects of the Δ*Cfatg4* mutant. Consequently, these recoveries resulted in the restoration of the defects of the Δ*Cfatg4* mutant in growth and responses to different external stresses, albeit to an extent. Importantly, we illustrated the critical roles of CfAtg8^1–160^ in appressoria formation, and pathogenicity. Collectively, our findings provide new insights into the importance of the interaction between CfAtg8 and CfAtg4 in the growth, autophagy and pathogenicity of the phytopathogenic fungi.

## 1. Introduction

*Camellia oleifera* is a Chinese native woody plant that is widely planted in southern China [1]. The tea oil extracted from its seed is highly appreciated for its rich unsaturated fatty acids and for use as raw materials in the cosmetics industry, contributing to the health and economic benefits [2,3]. However, anthracnose occurs wherever *C. oleifera* is grown and greatly impacts the tea oil yield and quality. We previously found that *Colletotrichum fructicola* is the dominant pathogen of anthracnose [4], but its molecular mechanisms underlying pathogenicity remain poorly understood.

Over the past 20 years, accumulated evidence supported the critical roles in autophagy to pathogenicity in phytopathogenic fungi [5,6,7,8,9]. Recently, we also revealed that the autophagy-related proteins CfAtg8, CfAtg9, and CfAtg5 are crucial for the pathogenicity of *C. fructicola* [10,11], initially illustrating the importance of autophagy in the pathogenicity of *Colletotrichum* spp. However, the precise regulatory mechanism is unknown.

Autophagy is a conserved pathway for the degradation and recycling of proteins and organelles in eukaryotes [12,13]. Atg8, which serves as the core protein of autophagy, mediates the expansion, closure, and fusion of the autophagosome membrane during autophagy and is a key protein in the formation of autophagosomes [14]. The reversible Atg8-phosphatidylethanolamine (PE) conjugation on the autophagosome membrane is a hallmark event in the autophagic process [15]. The cysteine protease Atg4 mainly acts on the coupling system of Atg8-PE. The newly synthesized Atg8 was modified by Atg4, exposing the glycine residues at the carbon terminal, which is specifically bound to the amine group of phosphatidylethanolamines (PEs) to form the Atg8-PE conjugation system [16,17,18]. The conjugated autophagosomes were integrated into lysosomes or vacuoles to complete the degradation steps in autophagy [19]. In addition, Atg4 also cleaves the amide bond of Atg8-PE in conjugation with autophagosomes and recovers Atg8 for the next round of the conjugation reaction [20,21]. Consequently, the protease activity of Atg4 is essential for autophagy and is highly specific, and many studies have listed Atg4 as a prospective target for the development of autophagy-specific inhibitors [22].

In this paper, we demonstrated that CfAtg4 interacts with CfAtg8 to control autophagosome formation and the subsequent autophagy flux. And we also provided evidence showing that CfAtg4-mediated autophagy governs the growth, development, and pathogenicity of *C. fructicola*.

## 2. Results

### 2.1. CfAtg4 Interacts with CfAtg8 in C. fructicola

**Figure 1:**
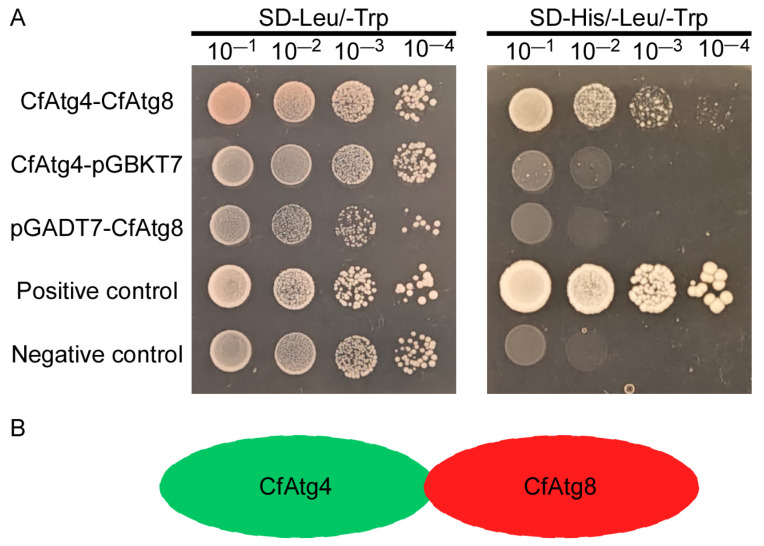
CfAtg4 interacts with CfAtg8. (**A**) The Y2H assays between CfAtg4 and CfAtg8. The pGADT7 and pGBKT7 vectors fused the related genes and were co-transformed into the yeast strain AH109. Leu: leucine; Trp: tryptophan; and His: histidine. (**B**) The schematic of the interaction between CfAtg4 and CfAtg8.

To demonstrate whether CfAtg4 interacts with CfAtg8 in *C. fructicola*, we conducted the yeast two-hybrid (Y2H) assay. The yeast cells carrying pGADT7-CfAtg4 and pGBKT7-CfAtg8 were grown on SD-His/-Leu/-Trp medium. In contrast, yeast carrying the empty vector and the negative control could not be grown on the same medium (Figure 1A,B). The results demonstrate that CfAtg4 interacts with CfAtg8 in *C. fructicola*.

### 2.2. The Acquisition of ΔCfatg4/CfAtg8^1–160^ Strains

**Figure 2:**
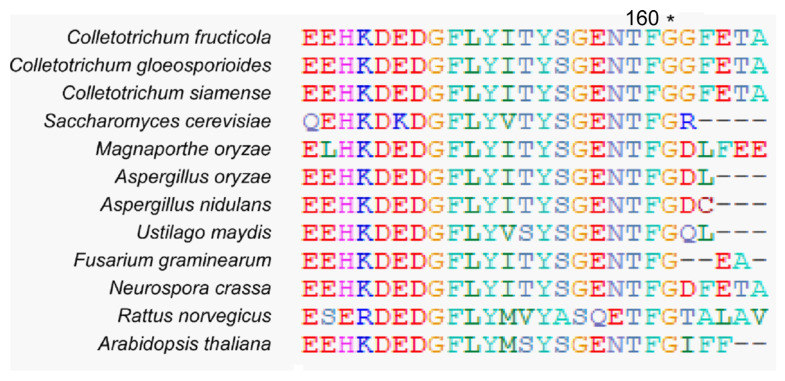
The alignment of Atg8 proteins among different species. The Atg8 proteins were aligned by the BioEdit v7.0 software and an asterisk indicates the conserved glycine of Atg8 proteins among fungi. The related species names are as follows: *C. fructicola*, *C. gloeosporioides*, *C. siamense*, *S. cerevisiae*, *M. oryzae*, *A. oryzae*, *A. nidulans*, *U. maydis*, *F. graminearum*, *N. crassa*, *R. norvegicus* and *A. thaliana*.

The cleavage of Atg8 in the carboxy-terminal glycine residue by Atg4 is a prerequisite for Atg8-PE formation and the latter autophagosome formation [20,23]. Accordingly, when the cleaved Atg8 instead of full-length Atg8 was expressed in the *ATG4* gene deletion mutant, the requirement for Atg8 cleavage could be bypassed [19]. Therefore, we identified the 160th glycine of CfAtg8 as the conserved cleavage site by the alignment of Atg8 proteins among different species using CLUSTAL_W programs (Figure 2). Then, we transferred a plasmid expressing the GFP-CfAtg8^1–160^ protein exposing glycine at site 160 to the Δ*Cfatg4* and obtained the Δ*Cfatg4*/CfAtg8^1–160^ strain.

### 2.3. CfAtg8^1–160^ Partially Restores Growth and Conidia Production of ΔCfatg4

**Figure 3:**
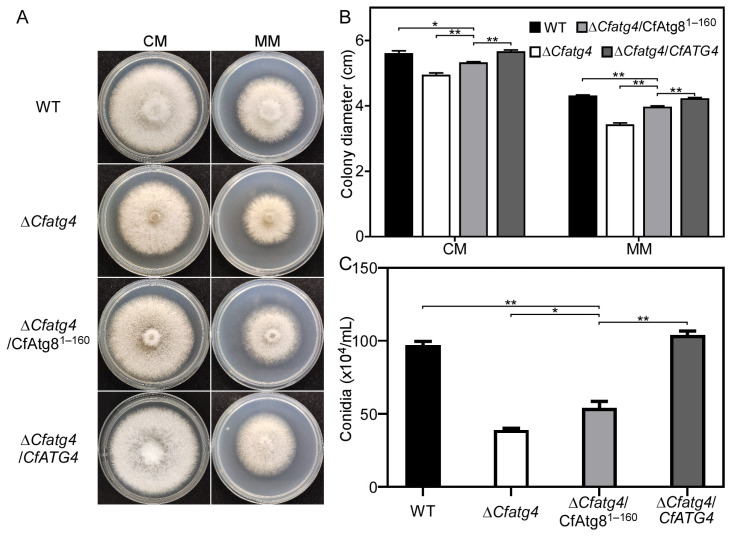
CfAtg8^1–160^ partially restores the growth and asexual reproduction of Δ*Cfatg4*. (**A**) The growth of WT, Δ*Cfatg4*, Δ*Cfatg4*/CfAtg8^1–160^, and Δ*Cfatg4*/*CfATG4* strains in CM and MM medium for 3 days. (**B**) The colony diameters were measured and statistically analyzed. (**C**) Statistical analysis of conidia. Asterisks mean the difference is significant (**, *p* < 0.01; *, 0.01 ≤ *p *< 0.05). The experiments were performed with 3 biological replications. The data were analyzed by Student’s *t*-test, and the error bars indicate standard deviation (SD).

To investigate whether Δ*Cfatg4*/CfAtg8^1–160^ could compensate for the growth defect of Δ*Cfatg4*, we cultured WT (wide-type), Δ*Cfatg4*, Δ*Cfatg4*/CfAtg8^1–160^, and complemented strain Δ*Cfatg4*/*CfATG4* on complete medium (CM) and minimal medium (MM) for 3 days. The results showed that the colony diameters of Δ*Cfatg4*/CfAtg8^1–160^ were significantly larger than those of the Δ*Cfatg4* strain but still significantly smaller than those of the WT strain and Δ*Cfatg4*/*CfATG4* strain on CM and MM media (Figure 3A,B). These results suggest that Δ*Cfatg4*/CfAtg8^1–160^ can partially compensate for the growth defect of Δ*Cfatg4*.

We next explored the roles of CfAtg8^1–160^ in conidia. By incubating WT, Δ*Cfatg4*, Δ*Cfatg4*/CfAtg8^1–160^, and Δ*Cfatg4*/*CfATG4* strains in liquid CM medium for 2 days, we found that Δ*Cfatg4*/CfAtg8^1–160^ showed a significant increase in conidia numbers compared to Δ*Cfatg4* but could not be fully restored to the level of WT, exhibiting only half of that to WT (Figure 3C). The results show that CfAtg8^1–160^ partially compensates for the conidia defects of Δ*Cfatg4*.

### 2.4. CfAtg8^1–160^ Partially Restores the Resistance of ΔCfatg4 to Rapamycin

**Figure 4:**
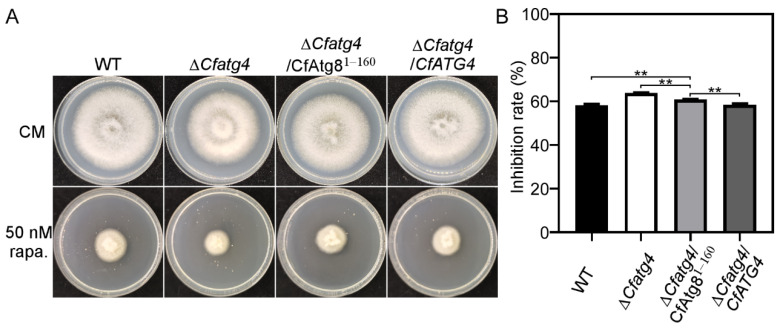
CfAtg8^1–160^ partially restores the resistance of Δ*Cfatg4* to rapamycin. (**A**) The WT, Δ*Cfatg4*, Δ*Cfatg4*/CfAtg8^1–160^, and Δ*Cfatg4*/*CfATG4* strains were incubated on CM plates with 50 nM of rapamycin at 28 °C for 3 days. (**B**) Statistical analysis of growth inhibition rates of the strains to rapamycin stress. The data were analyzed by Student’s *t*-test, and the error bars indicate SD. Asterisks indicate the difference is significant (**, *p* < 0.01).

Rapamycin is an agent that regulates autophagy by inhibiting the TOR kinase [24]. We inoculated WT, Δ*Cfatg4*, Δ*Cfatg4*/CfAtg8^1–160^, and Δ*Cfatg4*/*CfATG4* strains in CM medium supplemented with 50 nM rapamycin, which was used to initially explore the roles of CfAtg8^1–160^ in autophagy. The results showed that the Δ*Cfatg4*/CfAtg8^1–160^ strain was more tolerant compared to Δ*Cfatg4* (Figure 4A,B). However, the tolerance of the Δ*Cfatg4*/CfAtg8^1–160^ strain could not be restored to the level of the WT strain (Figure 4A,B). The above results indicate that CfAtg8^1–160^ makes Δ*Cfatg4* more resistant to rapamycin.

### 2.5. CfAtg8^1–160^ Is Important for Autophagy

**Figure 5:**
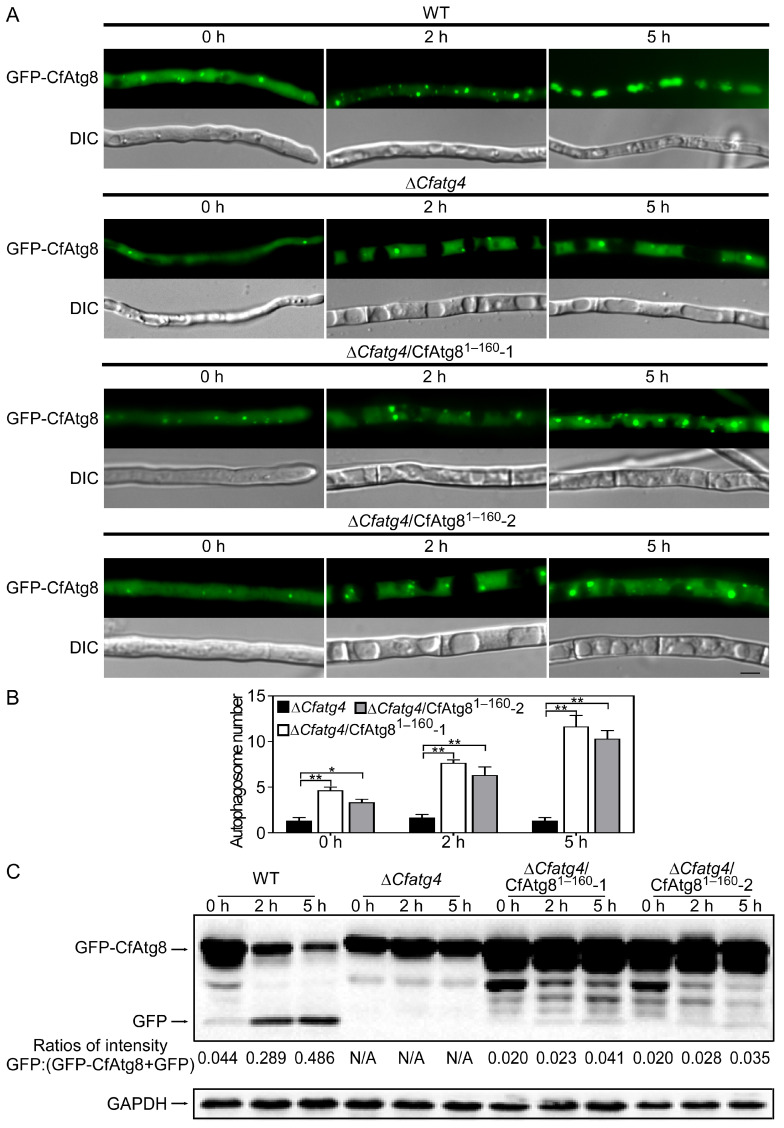
CfAtg8^1–160^ is important for autophagy. (**A**) Micrographs of GFP-CfAtg8-labeled autophagosomes in the WT, Δ*Cfatg4,* and Δ*Cfatg4*/CfAtg8^1–160^. (**B**) Statistical analysis of autophagosome number in Δ*Cfatg4* and Δ*Cfatg4*/CfAtg8^1–160^ after being induced for 0 h, 2 h, and 5 h in MM-N liquid medium. The data were analyzed by Student’s *t*-test, and the error bars indicate SD. Asterisks indicate the difference is significant (**, *p* < 0.01; *, 0.01 ≤ *p *< 0.05) (**C**) Immunoblot analysis of GFP-CfAtg8 proteolysis. The upper and lower lanes point to the intact GFP-Atg8 (46 kDa) and free GFP (26 kDa), respectively.

We acquired the Δ*Cfatg4* strain expressing GFP-CfAtg8^1–160^, and we further acquired the strains expressing GFP-CfAtg8 in the WT and Δ*Cfatg4* mutants, respectively. MM-N (minimal medium without NaNO_3_) treatment was another way to induce autophagy by inactivating TOR [25]. Similar to Δ*Cfatg4*, the GFP fluorescence of Δ*Cfatg4*/CfAtg8^1–160^ still could not be transferred into the vacuoles during MM-N-induced autophagy conditions compared with the GFP-contained vacuoles in WT (Figure 5A). However, the number of autophagosomes in Δ*Cfatg4*/CfAtg8^1–160^ was more than those in Δ*Cfatg4* before and after MM-N induction (Figure 5B). 

The process of autophagic flux was further tested by the subsequent breakdown of GFP-Atg8. After the autophagic body was transferred into the vacuoles, GFP-Atg8 was exposed to vacuolar hydrolases for degradation, whereas the intact GFP moiety cleaved from GFP-Atg8 was resistant to vacuolar proteolysis [26]. Thus, we can evaluate the autophagic flux by calculating the ratios of free GFPs relative to the total amount of intact GFP-CfAtg8 plus free GFP. We found that ratios were elevated as the induction time extended in WT expressing GFP-CfAtg8 and Δ*Cfatg4* expressing GFP-CfAtg8 strains showed no GFP for the whole time, whereas Δ*Cfatg4* expressing GFP-CfAtg8^1–160^ strains could produce free GFP despite a little increase (Figure 5C). The above results suggest that the cleavage of CfAtg8 by CfAtg4 is important for autophagy.

### 2.6. CfAtg8^1–160^ Partially Restores the Pathogenicity Defects of ΔCfatg4

**Figure 6:**
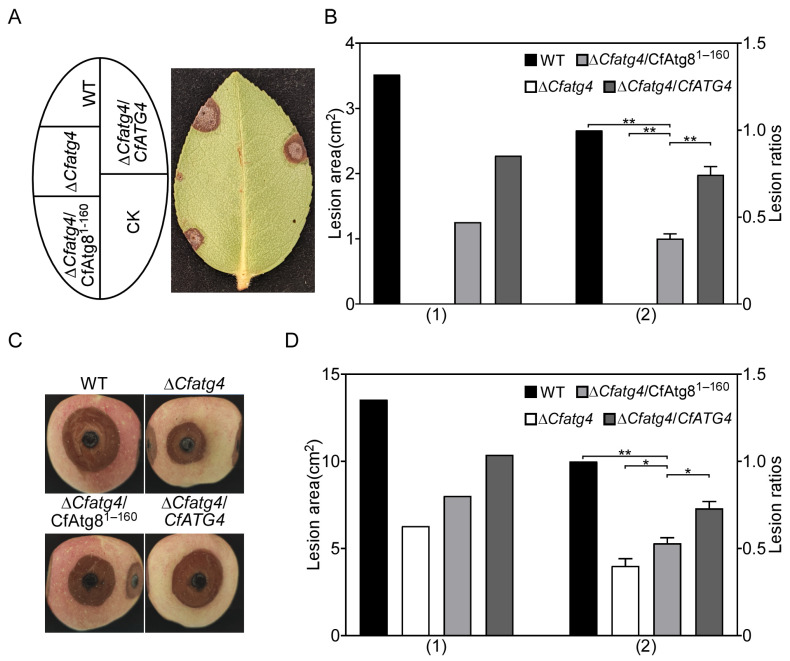
CfAtg8^1–160^ partially restores the pathogenicity of Δ*Cfatg4*. (**A**) The WT, Δ*Cfatg4*, Δ*Cfatg4*/CfAtg8^1–160^ and Δ*Cfatg4*/*CfATG4* strains were inoculated on wounded *C. oleifera* leaves. (**B**) The disease spot areas of strains on wounded *C. oleifera* leaves were measured by ImageJ, and the lesion ratios of the related strains to WT were statistically analyzed. (**C**) The strains were inoculated on wounded apples and photographed. (**D**) Statistical analysis of the related strains to WT on apples. CK: compared control. The data were analyzed by Student’s *t*-test, and the error bars indicate SD. Asterisks indicate the difference is significant (**, *p* < 0.01; *, 0.01 ≤ *p *< 0.05).

As CfAtg8^1–160^ partially restores autophagy, we speculate that it may also recover autophagy-related pathogenicity. The assay on the pathogenicity of wounded tea oil leaves showed that the Δ*Cfatg4*/CfAtg8^1–160^ strain restores the pathogenicity of the Δ*Cfatg4* mutant but could not conduct this to the same level as WT and Δ*Cfatg4*/*CfATG4* strains (Figure 6A,B). The pathogenicity assays were further tested on apples; the Δ*Cfatg4*/CfAtg8^1–160^ strain was inoculated on apples and showed significantly increased lesion areas compared to the Δ*Cfatg4* strain, and it was also unable to recover to the level of the WT strain (Figure 6C,D). The above results suggest that CfAtg8^1–160^ can partially restore the pathogenicity of Δ*Cfatg4*.

### 2.7. CfAtg8^1–160^ Partially Restores Conidia Germination and Appressoria Formation in ΔCfatg4

**Figure 7:**
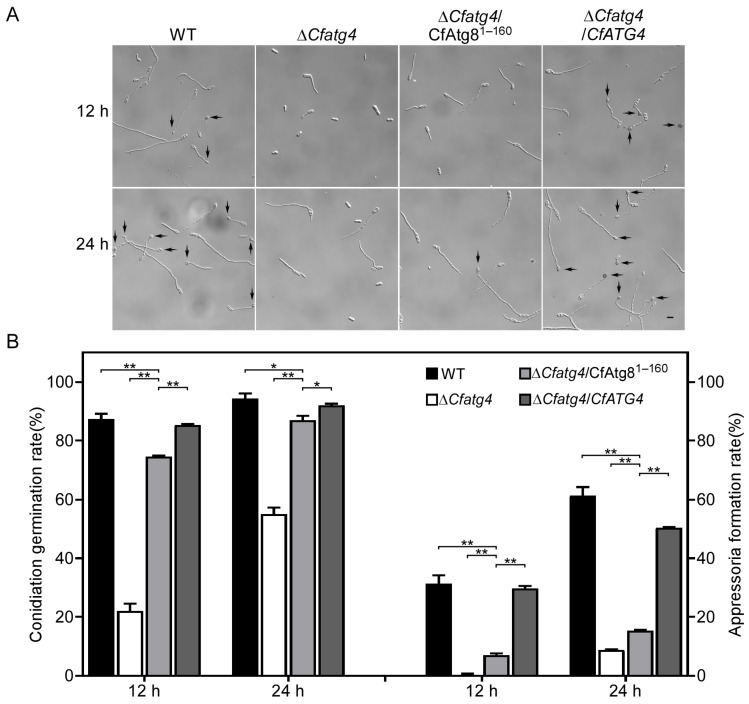
CfAtg8^1–160^ partially restores conidia germination and appressoria formation in Δ*Cfatg4*. (**A**) The conidia of the WT, Δ*Cfatg4*, Δ*Cfatg4*/CfAtg8^1–160^, and Δ*Cfatg4*/*CfATG4* strains were cultured on hydrophobic glass for 12 h and 24 h, and the appressoria formation was observed. (**B**) Conidia germination and appressoria formation were statistically analyzed. The data were analyzed by Student’s *t*-test and the error bars indicate SD. Asterisks indicate the difference is significant (**, *p* < 0.01; *, 0.01 ≤ *p* < 0.05). Arrows represent appressoria. Bar = 5 μm.

The appressoria of *Colletotrichum* spp. play critical roles in infesting hosts [27]. To explore the underlying mechanism of the partially recovered pathogenicity, the functions of CfAtg8^1–160^ in appressoria formation were examined. Therefore, we examined conidial germination and the appressoria formation of WT, Δ*Cfatg4*, Δ*Cfatg4*/CfAtg8^1–160^, and Δ*Cfatg4*/*CfATG4* after 12 and 24 h of incubation on hydrophobic glass slides. The results showed a significant increase in the germination rates of Δ*Cfatg4*/CfAtg8^1–160^ compared to Δ*Cfatg4*, but which was still significantly lower than those in WT and Δ*Cfatg4*/*CfATG4* (Figure 7A,B). A similar partial recovery was also observed in the appressoria formation among the strains above (Figure 7A,B). These results suggest that CfAtg8^1–160^ partially restores conidia germination and appressoria formation.

### 2.8. CfAtg8^1–160^ Helps ΔCfatg4 Respond to External Environmental Stresses

**Figure 8:**
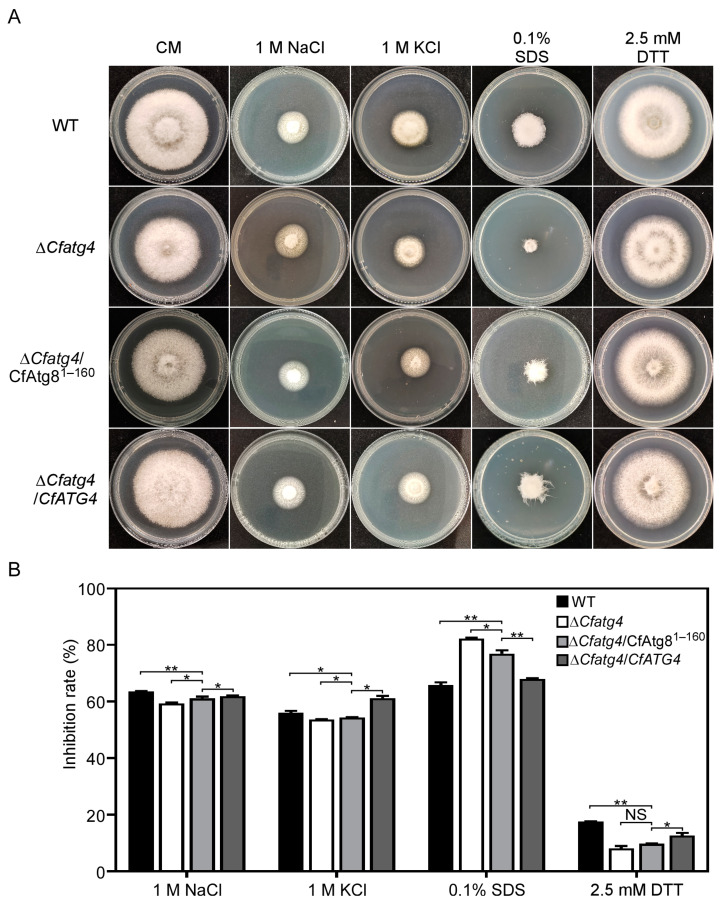
CfAtg8^1–160^ helps Δ*Cfatg4* respond to external environmental stresses. (**A**) The strains of the WT, Δ*Cfatg4*, Δ*Cfatg4*/CfAtg8^1–160^, and Δ*Cfatg4*/*CfATG4* were cultured on CM and CM plus 1 M of NaCl, 1 M of KCl, 0.1% SDS, and 2.5 mM of DTT for 3 days. (**B**) The inhibition rate of strains to stresses was statistically analyzed against an untreated control. The data were analyzed by Student’s *t*-test, and the error bars indicate SD. Asterisks indicate the difference is significant (**, *p* < 0.01; *, 0.01 ≤ *p* < 0.05).

During the interaction between pathogen and micro-environments, the cell wall, as the first barrier to contact with the outside world, is subject to external environmental stresses [28]. Furthermore, pathogens also face host-derived ER stress during infection [29,30]. Thus, we used several reagents to mimic the cell wall stress and ER stress. We inoculated WT, Δ*Cfatg4*, Δ*Cfatg4*/CfAtg8^1–160^, and Δ*Cfatg4*/*CfATG4* on the CM medium with 1 M NaCl, 1 M KCl, 0.1% SDS, and 2.5 mM DTT to explore the importance of Δ*Cfatg4*/CfAtg8^1–160^ in response to different environmental stresses. The results showed that CfAtg8^1–160^ partially recovers the osmotic stress (1 M NaCl and 1 M KCl) and cell wall stress (0.1% SDS) defects of the Δ*Cfatg4* mutant, while the inhibition rates showed no significant difference between Δ*Cfatg4* and Δ*Cfatg4*/CfAtg8^1–160^ on the ER stress (2.5 mM DTT) (Figure 8A,B). These results suggest that CfAtg8^1–160^ helps Δ*Cfatg4* respond to external environmental stresses.

## 3. Discussion

In our previous study, we initially found that CfAtg8-mediated autophagy regulates the growth, conidia production, appressoria formation, and pathogenicity of *C. fructicola* [11]. However, the molecular mechanisms underlying the regulatory roles of CfAtg8-mediated autophagy on pathogenesis remain largely unknown. Here, we revealed that CfAtg4 interacts with CfAtg8 to regulate the formation of autophagosomes and the autophagy-mediated pathogenicity in *C. fructicola*.

Through the yeast two-hybrid assay, we demonstrated that CfAtg4 interacts with CfAtg8 directly, which is consistent with studies performed using Atg4 and Atg8 orthologs in *S. cerevisiae*, *A. thaliana*, and *Sordaria macrospora* [20,31,32]. The study of *M. oryzae* further revealed the interaction between MoAtg4 and MoAtg8 under autophagy-induced conditions in vivo [33]. Thus, we hypothesized that Atg4 proteins share conserved functions in autophagy among different organisms.

In *S. cerevisiae* and *S. macrospora*, Atg8 proteins were C-terminally cleaved by Atg4 to generate Atg8-PE, mediating the autophagosome formation [20,23,32]. If Atg4 proteins share conserved roles in autophagy, CfAtg4 functions when autophagy occurs in *C. fructicola*. The targeted gene deletion of *CfATG4* caused a defect in autophagosome formation and autophagy flux, which is in line with the studies in *S. cerevisiae*, *M. oryzae*, and *Fusarium verticillioides* [33,34,35]. Moreover, we identified the 160th glycine of CfAtg8 as the conserved cleavage site and constructed an Atg8 variant GFP-CfAtg8^1–160^. When GFP-CfAtg8^1–160^ was introduced into the Δ*Cfatg4* mutant, it partially restored the response defects to rapamycin stress, which forecasted the roles of CfAtg8^1–160^ in autophagy. As expected, GFP-CfAtg8^1–160^ partially restores the formation of autophagosomes. Furthermore, the immunoblot analysis also revealed a slightly higher autophagy level in Δ*Cfatg4*/CfAtg8^1–160^ than that in the Δ*Cfatg4* mutant, demonstrating its partial recovery in the autophagy flux. It can be considered that except for the roles in the lipidation of Atg8, Atg4 also mediates the deconjugation of Atg8-PE to maintain a proper supply of Atg8 and facilitates the maturation of autophagosomes [19,35,36]. Consequently, though CfAtg8^1–160^ bypasses the cleavage steps, it is still defective in the deconjugation of CfAtg8-PE, resulting in defects in autophagy.

Nevertheless, CfAtg8^1–160^ partially restores the autophagy flux, which might foretell its roles in pathogenicity. Indeed, the lesions caused by CfAtg8^1–160^ are significantly larger than those caused by the Δ*Cfatg4* mutant, but still lesser than the typical lesions caused by WT and Δ*Cfatg4*/*CfATG4* strains. We reason that the partial recovery in the pathogenicity defects of CfAtg8^1–160^ is directly caused by the increased conidia germination and appressoria formation, which are important infectious processes for *Colletotrichum* to infect host plants [27,37,38]. Furthermore, based on the fact that the cell wall is the first barrier for fungi facing the host [28,30,39,40] and that the Δ*Cfatg4*/CfAtg8^1–160^ strain shows a slight recovery for the cell wall stresses, we concluded that this might also be a reason for its partial recovery in the pathogenicity. It might be very interesting to investigate how CfAtg4-mediated autophagy governs pathogenicity through conidia germination, appressoria formation, and cell wall stress responses. Thus, further studies are highly warranted.

In conclusion, our study not only illustrates the importance of the interaction between CfAtg4 and CfAtg8 in autophagy but also sheds light on the pleiotropic functions of the CfAtg4-mediated autophagy in conidiation germination, appressoria formation, cell wall stress responses, and the pathogenicity of *C. fructicola*. Therefore, we reveal that CfAtg4 is a prospective target in the development of new fungicides for anthracnose control.

## 4. Materials and Methods

### 4.1. Strains and Culture Conditions

In this study, CFLH16 was used as the wild type (WT). The *CfATG4* gene deletion mutant Δ*Cfatg4* and the complemented strains were obtained in our unpublished Chinese article. Briefly, two 1.0 kb of sequences flanking the *CfATG4* gene were PCR-amplified and overlapped to the flanks of the hygromycin resistance cassette (1.4 kb), respectively. After sequencing validation, the ~3.4 kb fragment was transformed into the protoplasts of WT to obtain the Δ*Cfatg4* mutant. The complement fragment, which contains the entire *CfATG4* gene and its native promoter region, was amplified by a PCR and inserted into pYF11 (bleomycin resistance) to complement the Δ*Cfatg4* mutant. Δ*Cfatg4*/CfAtg8^1–160^ was the strain expressing GFP-CfAtg8^1–160^ in the Δ*Cfatg4* mutant. For generating GFP-CfAtg8^1–160^, the GFP and the truncated CfAtg8 were amplified using the GFP-CfAtg8 plasmid as the template. Then, the fragment of GFP-CfAtg8^1–160^ was inserted into a pYF11 vector, as previously described [41].

All the strains were cultured in CM medium (10 g D-glucose, 2 g peptone, 1 g yeast extract, 1 g casamino acid, 1 mL vitamin solution [0.01 g biotin, 0.01 g pyridoxine, 0.01 g thiamine, 0.01 g riboflavin, 0.01 g p-aminobenzoic acid, and 0.01 g nicotinic acid in 1 L ddH_2_O), 1 mL trace elements [1.1 g H_3_BO_3_, 2.2 g ZnSO_4_•7H_2_O, 0.5 g FeSO_4_•7H_2_O, 0.5 g MnCl_2_•4H_2_O, 0.17 g CoCl_2_•6H_2_O, 0.15 g Na_2_MnO_4_•2H_2_O, 0.16 g CuSO_4_•5H_2_O, and 5 g Na_4_EDTA in 100 mL ddH_2_O, pH5.8], and 50 mL 20× nitrate salts [120 g NaNO_3_, 10.4 g MgSO_4_•7H_2_O, 10.4 g KCl, and 30.4 g KH_2_PO_4_ in 1 L ddH_2_O]) at 28 °C in dark conditions. The liquid CM was used to prepare mycelia for DNA and protein extraction. The MM-N medium (1.52 g KH_2_PO4, 0.52 g KCl, 0.152 g MgSO_4_·7H_2_O, 0.01 g vitamin B1, 1 mL trace elements, and 10 g D-glucose in 1 L of distilled water) was used to induce autophagy.

### 4.2. The Alignment of Atg8 Proteins

The Atg8 proteins in *C. fructicola*, *C. gloeosporioides*, *C. siamense*, *S. cerevisiae*, *M. oryzae*, *A. oryzae*, *A. nidulans*, *U. maydis*, *F. graminearum*, *N. crassa*, *R. norvegicus* and *A. thaliana* were obtained from the NCBI database. Then, the proteins were aligned using CLUSTAL_W programs by BioEdit v7.0 software.

### 4.3. Yeast Two-Hybrid Assays

The yeast two-hybrid assay was performed according to the instructions of the Alkali-Cation™ Yeast Transformation Kit (Mpbio, Santa Ana, CA, USA). The coding sequences of each candidate gene were amplified from the cDNA library of WT and then inserted into the bait vector pGBKT7 digested by BamHI and prey vector pGADT7 digested by BamHI, respectively, using the one-step ligase of Novozymes. After sequencing, pairs of plasmids were co-transformed into the yeast strain AH109 and then cultured on an SD-Leu-Trp medium. After growing colonies for 4 days, it was transferred on an SD-His/-Leu/-Trp medium. The positive control is ADRECT + BD (+), and the negative control is ADRECT + BD (−).

### 4.4. Growth, Conidiation and Appressoria Formation Assays

Small mycelial blocks of WT, ∆*Cfatg4*, ∆*Cfatg4*/*CfATG4,* and Δ*Cfatg4*/CfAtg8^1–160^ were cut from the edge of 3-day-old cultures and were cultured on CM and MM media for 3 days in the dark at 28 °C. Then, the diameters of the strains were measured and photographed. In the conidia production experiments, the strains above were cultured on a liquid CM in a shaker for 48 h, and the conidia were observed after filtering with three layers of filter paper. For appressoria formation assays, the collected conidia were dropped onto hydrophobic glass slides and incubated at 28 °C; then, they were observed and photographed under the microscope (ZEISS, Axio Observer. A1, Jena, Germany) at 12 h and 24 h. The germ tubes represent conidial germination, and the dome-shaped cells represent appressoria formation.

### 4.5. Stress Response Assays

The strains of WT, ∆*Cfatg4*, ∆*Cfatg4*/*CfATG4* and Δ*Cfatg4*/CfAtg8^1–160^ were cultured on a CM and CM medium with 50 nΜ of rapamycin, osmotic stress (1 M NaCl, 1 M KCl), endoplasmic reticulum stress (2.5 mM DTT) and cell wall integrity stress (0.1% SDS) for 3 days; the colony diameters were measured, and the inhibition rates were statistically analyzed. 

### 4.6. Pathogenicity Assays

The WT, ∆*Cfatg4*, ∆*Cfatg4*/*CfATG4,* and Δ*Cfatg4*/CfAtg8^1–160^ strains were inoculated on the edge of injured tea oil leaves in a moisturizing culture at 28 °C for 2 days; then its lesions were observed and measured. The above strains were also inoculated on wounded apples, as in our previous description [42]. First, several 4 mm diameter holes were punched evenly on the apples; then, strains of the same diameter were inoculated onto them. Finally, the apples were cultured in an incubator for 4~5 days, and the lesions were measured and statistically analyzed.

### 4.7. Autophagy Induction and Western Blotting Assays

The WT strains that transformed the GFP-CfAtg8 and the ∆*Cfatg4* strains and which transformed the GFP-CfAtg8 or GFP-CfAtg8^1–160^ strains were cultured on a liquid CM medium for 36 h; then the mycelia were washed with ddH_2_O and collected, followed by treatment with MM-N for 2 h or 5 h. The mean autophagosome numbers were calculated from at least 25 hyphae under a fluorescence microscope (ZEISS, Axio Observer. A1) and statistically analyzed. 

The Western blotting analyses were carried out according to our previous description [10]. First, the mycelia of the above strains, treated with CM and MM-N, were frozen in liquid nitrogen before protein extraction. Then, the frozen mycelia were ground into a fine powder in liquid nitrogen and re-suspended in a 1 mL lysis buffer (EpiZyme, PC101, Shanghai, China) with a 10 μL proteinase inhibitor cocktail (EpiZyme, GRF101). For protein lysing, the lysates were placed in the ice for 30 min before re-suspending with a vortex-genie once every 10 min, followed by centrifugation at 12,000 rpm for 20 min at 4 °C, and the supernatant proteins were harvested. The proteins were analyzed by 10% SDS-PAGE and Western blotting. The primary antibodies of protein analysis by Western blotting were anti-GFP (rabbit, 1:5000, Abways, AB0045) and anti-GAPDH (mouse, 1:20,000, jiahebio, M070101), and the second antibodies were HRP-labeled goat anti-rabbit lgG (H + L) (1:10,000, Abways, AB0101) or HRP-labeled goat anti-mouse lgG (H + L) (1:20,000, HUABIO, HA1006). After that, the proteins were detected by the Omni-ECL Femto Light Chemiluminescence kit (EpiZyme, SQ201) and analyzed by ImageJ v1.48.

### 4.8. Accession Number

The sequence data of this research can be acquired in the GenBank database (https://www.ncbi.nlm.nih.gov/genbank/, accessed on 20 February 2023) with the following accession numbers: KAF4481970.1 (CfAtg8), XP_045259742.1 (CgAtg8), XP_036500530.1 (CsAtg8), NP_009475.1 (ScAtg8), KAI6350462.1 (MoAtg8), XP_001727929.1 (AoAtg8), XP_662735.1 (AnAtg8), XP_011391873.1 (UmAtg8), XP_011325668.1 (FgAtg8), XP_956248.1 (NcAtg8), 1KJT_A (RnAtg8) and AAM64870.1 (AtAtg8).

### 4.9. Statistical Analysis

All data are presented as the mean ± standard deviation and analyzed by Student’s *t*-test, *p* < 0.01 or 0.01 ≤ *p* < 0.05.

## Data Availability

The original contributions presented in the study are included in the article, further inquiries can be directed to the corresponding author.
